# Evaluating the effectiveness of measures to control the novel coronavirus disease 2019 in Jilin Province, China

**DOI:** 10.1186/s12879-021-05936-9

**Published:** 2021-03-06

**Authors:** Qinglong Zhao, Yao Wang, Meng Yang, Meina Li, Zeyu Zhao, Xinrong Lu, Bo Shen, Bo Luan, Yifei Zhao, Bonan Cao, Laishun Yao, Benhua Zhao, Yanhua Su, Tianmu Chen

**Affiliations:** 1 Jilin Provincial Center for Disease Control and Prevention, Changchun, Jilin Province 130062 People’s Republic of China; 2 State Key Laboratory of Molecular Vaccinology and Molecular Diagnostics, School of Public Health, Xiamen University, 4221-117 South Xiang’an Road, Xiang’an District, Xiamen City, Fujian Province 361102 People’s Republic of China; 3 The First Hospital of Jilin University, Changchun, Jilin Province 130021 People’s Republic of China

**Keywords:** COVID-19, Epidemic, Measures, Transmissibility

## Abstract

**Background:**

Based on differences in populations and prevention and control measures, the spread of new coronary pneumonia in different countries and regions also differs. This study aimed to calculate the transmissibility of coronavirus disease 2019 (COVID-19), and to evaluate the effectiveness of measures to control the disease in Jilin Province, China.

**Methods:**

The data of reported COVID-19 cases were collected, including imported and local cases from Jilin Province as of March 14, 2019. A Susceptible–Exposed–Infectious–Asymptomatic–Recovered/Removed (SEIAR) model was developed to fit the data, and the effective reproduction number (*R*_*eff*_) was calculated at different stages in the province. Finally, the effectiveness of the measures was assessed.

**Results:**

A total of 97 COVID-19 infections were reported in Jilin Province, among which 45 were imported infections (including one asymptomatic infection) and 52 were local infections (including three asymptomatic infections). The model fit the reported data well (*R*^2^ = 0.593, *P* < 0.001). The *R*_*eff*_ of COVID-19 before and after February 1, 2020 was 1.64 and 0.05, respectively. Without the intervention taken on February 1, 2020, the predicted cases would have reached a peak of 177,011 on October 22, 2020 (284 days from the first case). The projected number of cases until the end of the outbreak (on October 9, 2021) would have been 17,129,367, with a total attack rate of 63.66%. Based on the comparison between the predicted incidence of the model and the actual incidence, the comprehensive intervention measures implemented in Jilin Province on February 1 reduced the incidence of cases by 99.99%. Therefore, according to the current measures and implementation efforts, Jilin Province can achieve good control of the virus’s spread.

**Conclusions:**

COVID-19 has a moderate transmissibility in Jilin Province, China. The interventions implemented in the province had proven effective; increasing social distancing and a rapid response by the prevention and control system will help control the spread of the disease.

## Background

Coronavirus disease 2019 (COVID-19) is caused by SARS-CoV-2 with typical symptoms of fever, dry cough, and tiredness [1–3]. On average, the incubation period is 5–6 days from the time someone is infected with the virus to the onset of symptoms, with a maximum of 14 days [3]. Nucleic acid detection and genome sequencing have commonly been conducted with pharyngeal swabs, sputum, alveolar lavage fluid, feces, and other samples from patients to detect SARS-CoV-2 [4–8]. It has been reported that COVID-19 can be transmitted person-to-person, with the main transmission methods being either by air or contact [9–13]. Therefore, persons can be infected by inhaling droplets or aerosols containing the etiologic agent SARS-CoV-2 that are exhaled by someone with the infection, or by contacting virus-contaminated items.

The World Health Organization (WHO) announced that this disease represented a public health emergency of international concern. Due to its diverse transmission routes and strong transmissibility, COVID-19 quickly became pandemic. As of April 8, the number of confirmed cases worldwide reached 1,353,361 and there were 79,235 cumulative deaths [14]. According to the report of the Chinese Health Commission, as of April 9, a total of 81,865 confirmed cases and a total of 3335 deaths were reported in China [15]. Since Jilin Province launched the Public Health Events level I emergency response on January 25, the epidemic in Jilin Province has been controlled by implementation of measures to control the non-resident population, such as isolation and observation at home, temperature measurement screening, and wearing masks [16]. According to data from the Jilin Provincial Center for Disease Control and Prevention, as of March 14, a total of 97 cases with one death were reported [17]. Although the severity of the domestic epidemic has declined, the problems of imported cases and asymptomatic cases remain serious.

Several studies of COVID-19 transmission models have been conducted to evaluate the transmissibility of the virus and predict the future pandemic situation [9, 18–20]. In COVID-19 transmission models, the influence of asymptomatic infection factors in the transmission process is considered. This study is based on our previous research, with the addition of an asymptomatic infection factor. We use the epidemic data of Jilin Province to re-verify the applicability of the susceptible–exposed–infectious–asymptomatic–recovered/removed (SEIAR) model, and to further discuss the role of asymptomatic infection in the spread of COVID-19 [21–24]. The more important issue at present is to consider asymptomatic infections when designing models. Asymptomatic infection refers to cases who tested positive for COVID-19 in laboratory tests and had no symptoms, but can still potentially transmit the virus to others. In a report in Nature on March 20, 2020, a public health expert from Wuhan Huazhong University of Science and Technology noted that “at least 59% of the infected individuals were out and about, without being tested and potentially infecting others” [25]. The calculated transmissibility results of a model will differ, depending on whether the model considers asymptomatic infection. That is, ignoring asymptomatic cases will affect the accuracy of the model. At the same time, traditional infectious disease models were built under the condition that the disease is allowed to develop [2, 9, 18, 19, 26–30]. However, China declared a first-level health emergency in the early stage of the outbreak, and, with a strict supervision system and a high degree of cooperation of the people, a series of prevention and control measures were implemented, such as wearing masks, restricting travel, and suspending work and school. In this study, our COVID-19 model was established with thorough consideration of most of the possible comprehensive prevention and control measures that exist. Moreover, there is no domestic province that can be used to construct a dynamic model of the spread of COVID-19 according to the local population characteristics and distribution. Hence, the transmissibility of COVID-19 in Jilin Province remains unclear and the effect of current prevention and control measures on the pandemic still needs to be explored. This study focused on the SEIAR model based on the distribution of outbreaks in Jilin Province. The various parameters in the model were calculated based on the actual cases obtained, to accurately model the real situation. This study explored the goodness of fit between the model and actual data, calculated the transmissibility of COVID-19 in Jilin Province, and evaluated the effectiveness of local health departments’ prevention and control measures. We further predicted the progress of theCOVID-19 pandemic if no measures were taken at the corresponding time point or if intervention measures were implemented at different time points.

## Methods

**Fig. 1:**
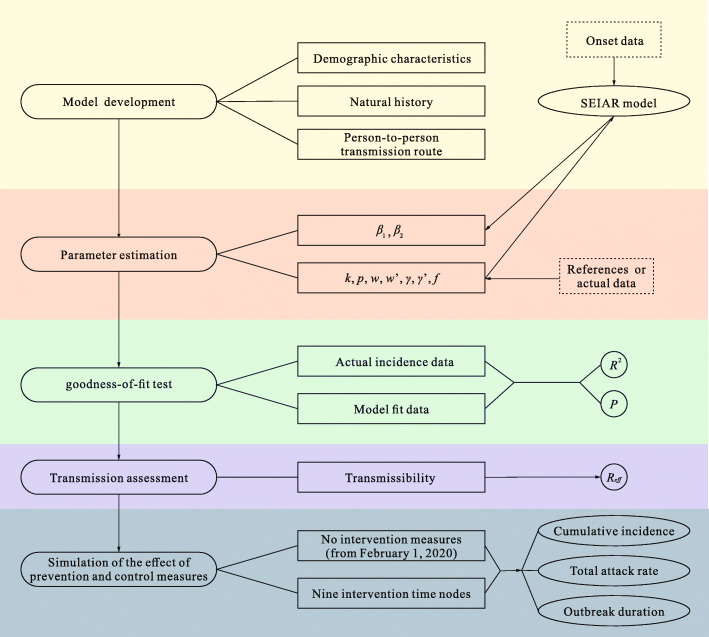
Research technical route

This research was carried out in sequence according to the five steps of model development, parameter estimation, model effectiveness evaluation, transmission assessment, and simulation of the effects of prevention and control measures (Fig. 1). First, parameters were set and the SEIAR model established based on the collected demographic characteristics, natural history of the disease, and person-to-person transmission route. References and actual data were used to calculate parameters consistent with the actual COVID-19 situation in Jilin Province. The inflection point of the pandemic (February 1, 2020) was set as the intervention time node, case data by onset date were obtained, and data were substituted into the model equations to obtain fit parameters. The model fit was compared with actual onset data to calculate the goodness of fit. The *β* value obtained by model fitting was substituted into the formula for calculating *R*_*eff*_ to obtain the transmissibility. The degree of transmissibility decline was calculated before and after the time node of the intervention measures. Finally, a simulation was carried out based on the assumption that no measures were taken in the segment time, to predict the duration and prevalence of the pandemic in that case. The progress of the pandemic was also estimated given intervention measures at nine different time points.

### Data collection

The case information collected in this article was provided by the Jilin Provincial Center for Disease Control and Prevention. The deadline for data collection was March 14, 2020, including onset date, diagnosis date, date of contact with related cases, clinical classification (based on the standards set by the National Health Commission of China in accordance with clinical symptoms) [31], and laboratory diagnosis of different case types. In addition, the permanent population of Jilin Province was obtained from the “Jilin Statistical Yearbook.”

### Transmission model

According to the COVID-19 propagation dynamic model that we built [21–24], the SEIAR model of “person-to-person” secondary cases of COVID-19 in Jilin Province extended from January 22 to February 19, 2020, with only 29 days of pandemic. Due to the short duration of COVID-19 in Jilin Province, the number of people who were born or died of natural causes during the epidemic period can be ignored. Therefore, compared with our previous SEIAR model, the model of the COVID-19 pandemic in Jilin Province ignored natural births and natural deaths. The model was based on the following assumptions:

1. The model divides the population into five categories: susceptible (*S*), exposed (*E*), infectious (*I*), asymptomatic (*A*), and recovered/removed (*R*).
2. Both *I* and *A* are infectious, and *A*’s transmissibility is *k* times that of *I* (0 < *k* ≤ 1). *S* may be infected when exposed to *I* and *A*, and the infection rate coefficient is *β*. Therefore, at time *t*, the infected *S* is *βS* (*I* + *A*).
3. Among *E*, the proportion of those who develop asymptomatic infections is *p*, the incubation period is 1/*ω*, and the latent period is 1/*ω’*. Then at time *t*, there are *pω’E* persons in *E* who develops into *A*, and (1- *p*)*ωE* persons become *I*. According to the tracking and observation of close contacts in previous studies [23], *E* is not contagious in Jilin Province, and is contagious only when it changes to *A* or *I*.
4. *I*, from onset to admission is 1/*γ* days; that is, there are *γ I* admitted to the hospital per unit time. Therefore, at time *t*, there are *γ I* people in *I* who change to movers. The case fatality rate of *I* is *f*; so, at time *t*, *f I* people die in *I*.
5. *A* has an infectious period of 1/*γ*’, that is, *γ*’ persons in *A* escape from the infectious period in unit time. Therefore, at time *t*, there are *γ*’ *A* people in *A* who are transformed into movers.

**Fig. 2:**
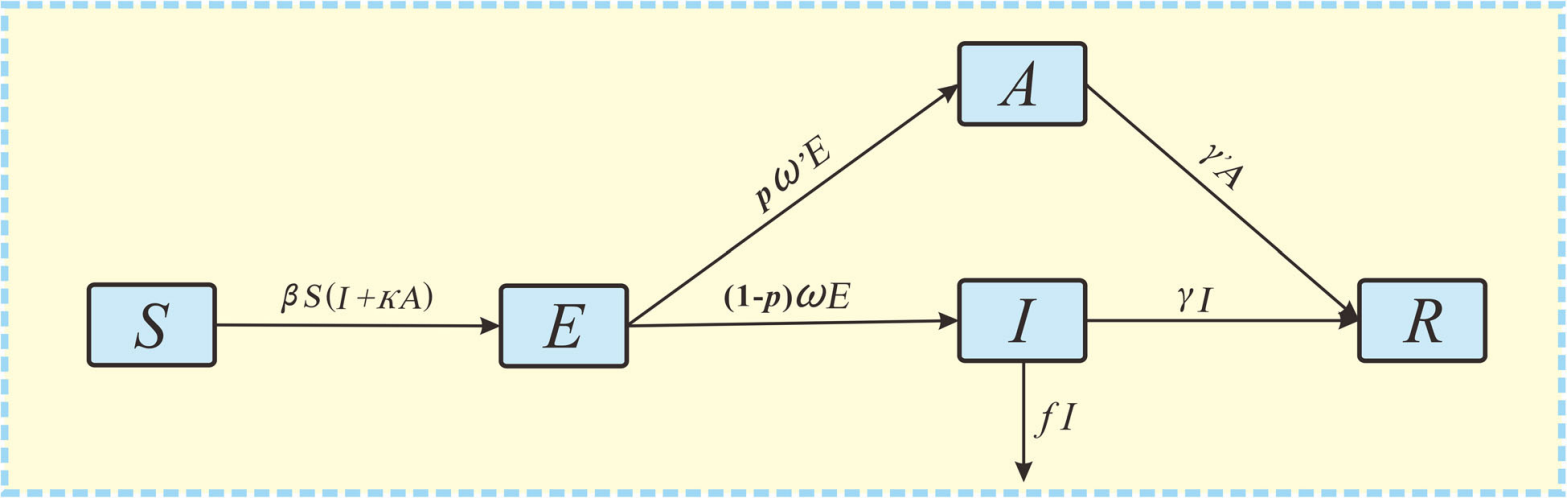
SEIAR model for simulating COVID-19

Therefore, the framework of the SEIAR model with the natural birth rate and mortality rate of the population removed is shown in Fig. 2. The differential equations of the model are as follows:

$$ {\displaystyle \begin{array}{l} dS/ dt=\hbox{-} \beta S\left(I+ kA\right)\\ {} dE/ dt=\beta S\left(I+ kA\right)- p\omega \hbox{'}E\hbox{-} \left(1-p\right)\omega E\\ {} dI/ dt= np+\left(1-p\right)\omega E-\gamma I\\ {} dA/ dt= p\omega \hbox{'}E-\gamma \hbox{'}A\\ {} dR/ dt=\gamma I+\gamma \hbox{'}A\end{array}} $$

### Parameter estimation

The total number of susceptible people was derived from the number of permanent residents in Jilin Province recorded in the Jilin Statistical Yearbook. According to the actual incidence characteristics of COVID-19 in Jilin Province, the cases were divided into two types: imported cases as the source of infection and secondary cases used as the actual data to fit the model. Since February 1, 2020, the trend of secondary cases over time reached an inflection point, and the number of daily incidences has shown a downward trend. This shows that under the prevention and control measures adopted by the relevant health departments in Jilin Province, the daily incidence has been significantly reduced. Therefore, the transmissibility of the disease was different before and after February 1; the infection rate coefficient *β* changes significantly before versus after February 1. Therefore, February 1 was set as the time segment node, and the infection rate coefficients (*β*1 and *β*2) were respectively obtained by model fitting. According to previous research by our team, the transmissibility of asymptomatic infections is the same as symptomatic infections, *k* = 1. There were four asymptomatic infections among 97 cases in Jilin Province; that is, the proportion of asymptomatic infections was 0.04. To calculate the time interval from infection to symptom onset in all cases in Jilin Province, except for asymptomatic infections, the median was calculated as 10. The previous literature showed that the latent period of asymptomatic infections is the same as that the incubation period of typical infections [21]; therefore, *ω* = *ω*’ = 0.1. The time interval from onset to admission of infectious cases in Jilin Province was calculated, and the median was 3. Because asymptomatic infections are mostly admitted to hospital for isolation treatment for intensive contacts, the number of infections, and the proportion of asymptomatic infections in Jilin Province are small, the period of infection of asymptomatic infections was similar to that of infections. Therefore, *γ* = *γ*’ = 0.33. According to the statistics on COVID-19 in Jilin Province, there was only one death among all patients. Therefore, in the COVID-19 model for the province, the mortality rate f was negligible, that is, *f* = 0. The model parameter values and methods are shown in Table 1.

**Table 1:** The definition and values of parameters in SEIAR model of COVID-19 in Jilin Province, China

Parameter	Description	Unit	Value	Parameter source
*β*_1_	Infection rate coefficient (before February 1)	Person^− 1^·day^− 1^	6.7865 × 10^− 9^	Curve Fitting
*β*_2_	Infection rate coefficient (after February 1)	Person^−1^·day^−1^	2.0519 × 10^− 10^	Curve Fitting
*k*	Coefficient of Transmissibility of A relative to *I*	1	1	literature [21]
*p*	Proportion of asymptomatic infections	1	0.04	Actual data
*ω*	Relative rate of incubation period of *I*	day^−1^	0.1	Actual data
*ω’*	Relative rate of latent period of *A*	day^−1^	0.1	literature [21]
*γ*	Coefficient of time between onset and admission	day^−1^	0.33	Actual data
*γ’*	Infection period coefficient	day^−1^	0.33	literature [21]
*f*	Fatality rate	1	0	Actual data

### Transmissibility of COVID-19

Under ideal circumstances, the basic reproduction number (*R*_0_) can be used to quantify the transmissibility of COVID-19 [3, 21, 32, 33]; *R*_0_ is the number of cases in which the source of infection directly spread the virus during the infection period. Comparing the *R*_0_ value with 1 can be used as an index to evaluate whether the disease is prevalent. If the evaluated disease does not spread in a natural state because of the use of isolation, vaccines, and other interventions, *R*_0_ cannot reflect the actual spread of the disease. At this time, an effective reproduction number (*R*_*eff*_) is needed to represent transmissibility. Based on previous research [34–36], *R*_*eff*_ can be expressed by the following equation:

$$ \underset{dr\to \infty }{\lim }{R}_{eff}=\beta S\left(\frac{1-p}{\gamma +f}+\frac{\upkappa p}{\gamma^{\prime }}\right) $$

At the same time, because the mortality rate of COVID-19 in Jilin Province is close to 0, the equation can be simplified to:

$$ {R}_{eff}=\beta S\left(\frac{1-p}{\gamma }+\frac{\upkappa p}{\gamma^{\prime }}\right) $$

### Simulation method and statistical analysis

The software Berkeley Madonna 8.3.18 was used to model the actual cases, and the fourth-order Runge-Kutta method was used to solve the differential equations. Curve estimation in SPSS 20.0 was used to compare the fitted data with the actual data, and to observe the *P* and *R*^2^ values to judge the goodness of fit.

## Results

### Epidemiological characteristics

**Fig. 3:**
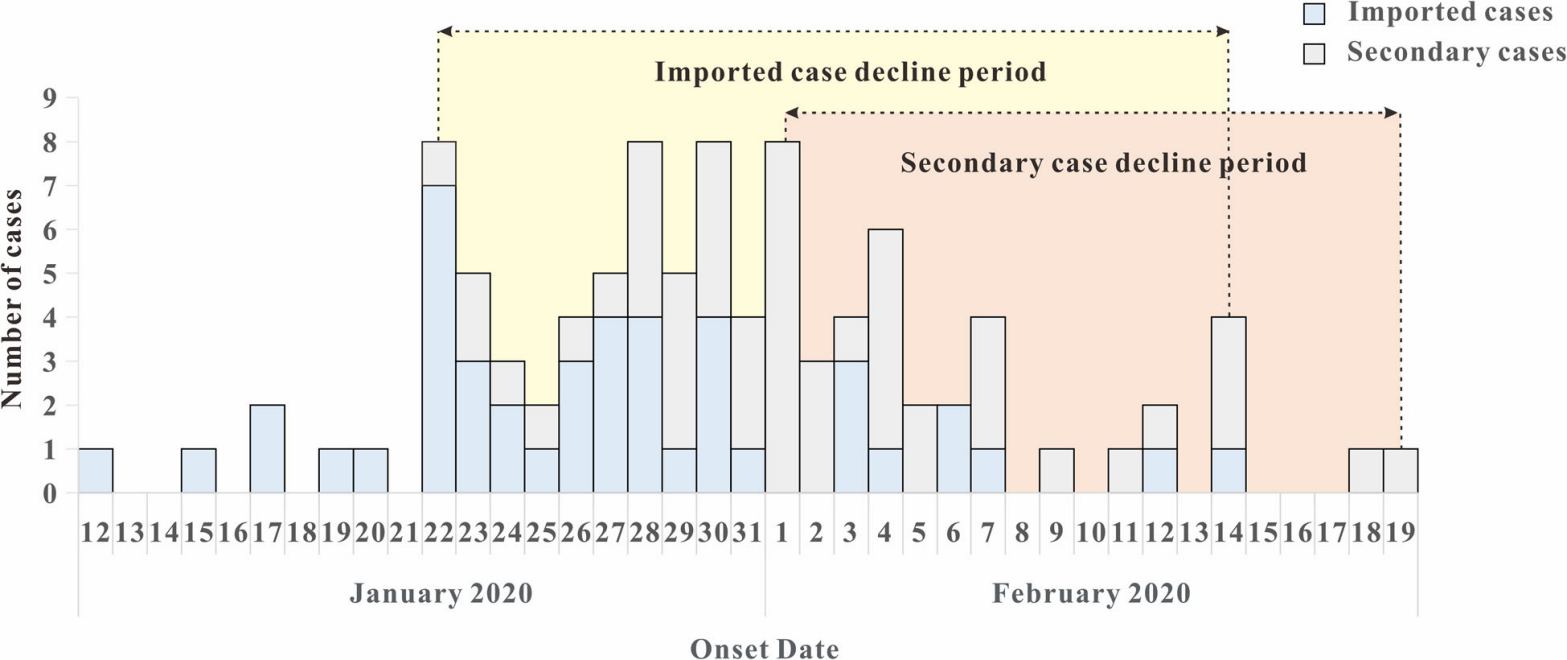
Temporal distribution of COVID-19 in Jilin Province, China

As of the date of data collection (March 14), from the first imported case on January 12 to the last case on February 19, there were 97 COVID-19 infections reported, namely 45 imported infections (including one asymptomatic infection) and 52 secondary infections (including three asymptomatic infections). The first case in Jilin Province was an imported case whose onset date was January 12, 2020. The onset date of the first secondary case was January 22, 2020, and local secondary cases were the main cases in the later stage of the pandemic. The peak date of the incidence of imported cases was January 22, and the peak of local cases was February 1. A stacked histogram of changes is shown in Fig. 3.

**Fig. 4:**
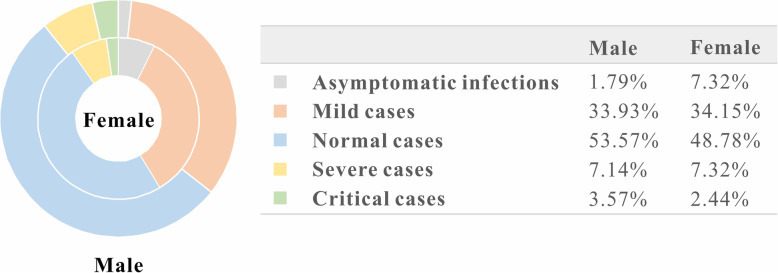
The proportion of disease severity according to gender in Jilin Province, China

Regarding the gender breakdown (Fig. 4), there were 56 males and 41 females. According to the clinical classification standards of the National Health Commission of China and the actual clinical classification data provided by the Jilin Provincial Center for Disease Control and Prevention, among male and female cases, normal cases predominated, accounting for 54 and 49% of all case types, respectively. In descending order, these were followed by mild, severe, and critical cases. There were slightly fewer asymptomatic infections in men than severe cases, and the number of asymptomatic infections among women is the same as the number of critical cases.

**Fig. 5:**
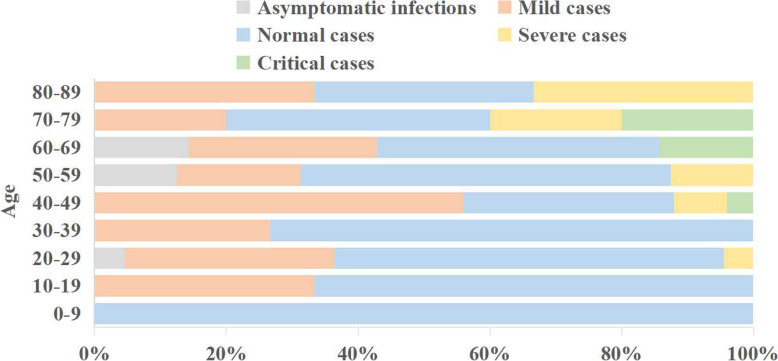
The proportion of disease severity in different age groups in Jilin Province, China

The proportion of disease severity of different age groups was analyzed (Fig. 5). The age of onset was concentrated between 20 and 59 years, accounting for 80.41% of the total number of patients. Among all reported cases, the proportion of mild cases in the 40–49 age group was 56%, the proportion of normal cases in the 0–9 age group was 100%, the proportion of severe cases in the 80–89 age group was 33.33%, the proportion of critical cases in the 70–79 age group was up to 20%, and the proportion of asymptomatic infections in the 60–69 age group was 14.29%. The proportion of normal cases was highest in different age groups, and the number of cases decreased as the severity of the disease increased.

### Model fitting and calculation of transmissibility

**Fig. 6:**
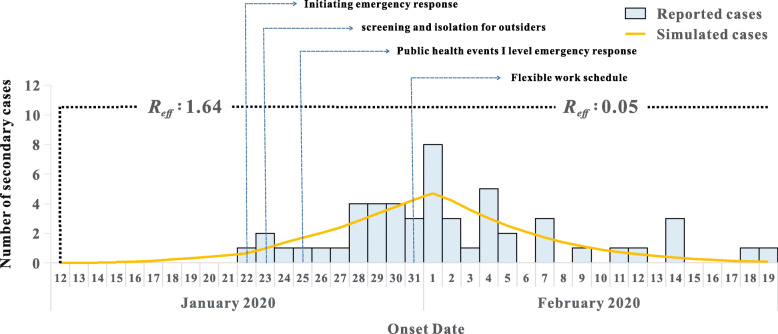
The fitting results of the SEIAR model and the data of the actual secondary cases of COVID-19 cases in Jilin Province, China

According to the comparison between the model fitting curve and the actual secondary cases curve (Fig. 6), the fit was good. At the same time, the goodness-of-fit test results showed that the difference between secondary cases fitted by the model and the actual secondary cases was statistically significant (*R*^2^ = 0.593, *P* < 0.001). The values of *β*_1_ and *β*_2_ obtained by the model fitting were brought into the formula for *R*_*eff*_. The *R*_*eff*_ of COVID-19 cases before February 1 was 1.64, the *R*_*eff*_ of COVID-19 cases after February 1 was 0.05; the transmissibility decreased by 96.95%.

**Fig. 7:**
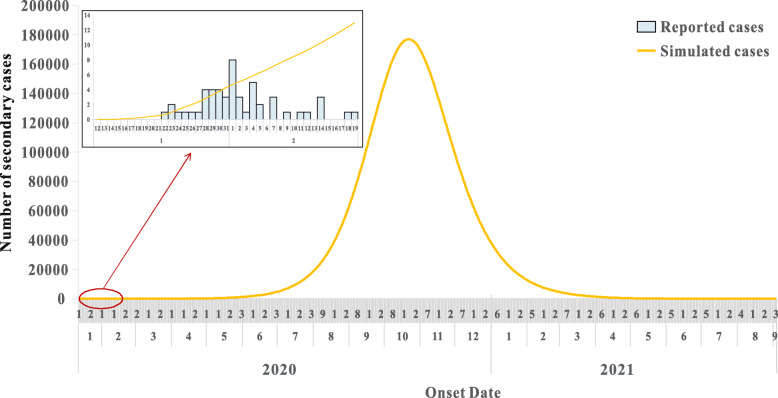
Simulation results of the SEIAR model without intervention and the data of the actual secondary cases of COVID-19 cases in Jilin Province, China

It is known that after February 1, the incidence of COVID-19 showed a downward trend, and the last case occurred on February 19 (Fig. 3). If no intervention measures had been taken after the onset of new coronary pneumonia, the model fit the curve of the future incidence in this scenario (Fig. 7). The model predicted that if no measures had been taken, the incidence on February 19 would have been 13 cases, while the actual incidence on that date was one case. Therefore, the comprehensive interventions reduced the incidence by 92.31%. If the epidemic situation had been allowed to continue, the incidence curve would have resembled a bell shape, with a peak on October 22, 2020 (284 days from the first case), with 177,011 cases on that day, and the pandemic would have lasted for 22 months. At the same time, the forecast also predicted the onset at the end of each month in the near future (Table 2). Without the interventions taken on February 1, 2020, a total of 17,129,367 cases would have been reported until the end of the outbreak (on October 9, 2021), with a total attack rate of 63.66%. These results reveal that the interventions implemented in Jilin Province reduced the number of cases by more than 99.99%.

**Table 2:** Prediction of the prevalence of COVID-19 in Jilin Province without comprehensive intervention measures on February 1, 2020

Date	Number of cases	Cumulative number of cases	Attack rate
Feb. 29, 2020	20	356	1.32E-05
Mar. 31, 2020	82	1755	6.52E-05
Apr. 30, 2020	313	7033	2.61E-04
May.31, 2020	1250	28,485	1.06E-03
Jun.30, 2020	4742	108,914	4.05E-03
Jul.31, 2020	18,217	427,535	1.59E-02
Aug.31, 2020	62,146	1,578,295	5.86E-02
Sept.30, 2020	144,375	4,674,584	1.74E-01
Oct.31, 2020	170,712	9,908,011	3.68E-01

**Fig. 8:**
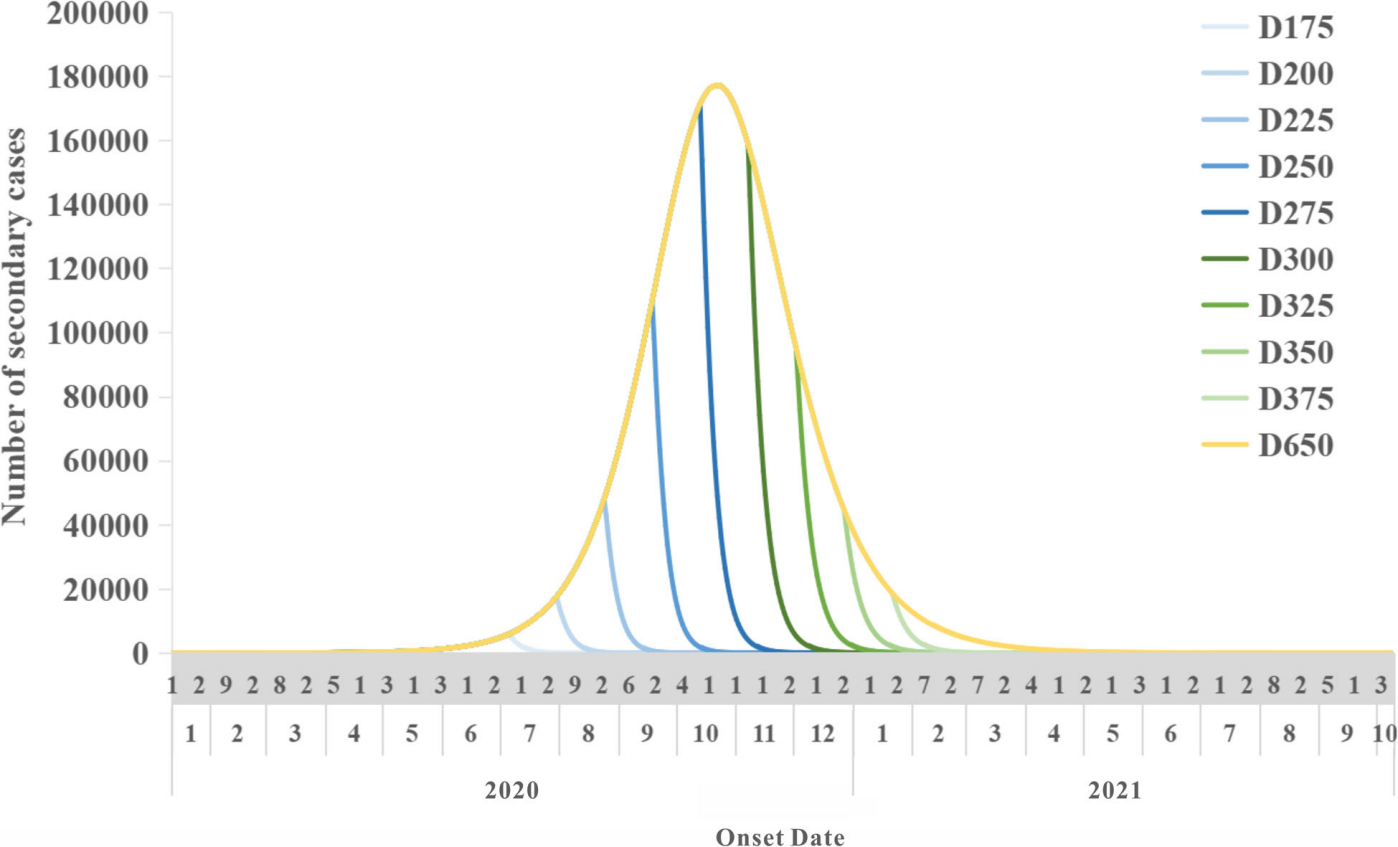
COVID-19 prevalence curve and peak incidence after taking measures at different time points in Jilin Province, China

In addition, epidemic curve and peak incidence of COVID-19 were predicted given implementation of measures at different time points. Figure 8 shows the future incidence curve when the number of days from the first case varied (175 days, 200 days, 225 days, 250 days, 275 days, 300 days, 325 days, 350 days, 375 days). The trend changed into a gradual decline in curve. The prevalence of measures taken at different time points shows that the sooner measures are taken, the more easily the outbreaks can be controlled, the lower the peak number of outbreaks, the earlier the end of the outbreak, and the lower the cumulative number of outbreaks (Table 3).

**Table 3:** Prediction of the prevalence of COVID-19 in Jilin Province after adopting comprehensive intervention measures at different time points after February 1, 2020

Time for comprehensive intervention	Cumulative number of cases	Attack rate	Peak date	Peak incidence	Outbreak duration
D175	170,197	0.63%	Jul. 5, 2020	5911	8 month
D200	510,848	1.90%	Jul. 30, 2020	17,462	9 month
D225	1,471,201	5.47%	Aug. 24, 2020	48,043	10 month
D250	3,805,023	14.14%	Sept. 18, 2020	109,787	11 month
D275	7,892,257	29.33%	Oct. 13, 2020	171,253	12 month
D300	12,193,645	45.32%	Oct. 22, 2020	177,011	13 month
D325	14,939,812	55.52%	Oct. 22, 2020	177,011	14 month
D350	16,235,738	60.34%	Oct. 22, 2020	177,011	15 month
D375	16,775,568	62.35%	Oct. 22, 2020	177,011	16 month
D650	17,129,367	63.66%	Oct. 22, 2020	177,011	22 month

## Discussion

Based on the epidemic situation of COVID-19 in Jilin Province, we constructed a transmission dynamics model that accorded with the population characteristics of the province. Furthermore, based on the collection of 97 cases as of March 14, the true parameters of Jilin Province were calculated. Using imported cases as the source of infection, the model calculated fitted secondary cases based on local secondary cases. Therefore, the design of the model, the calculation of parameters and the fitting of data were consistent with the actual situation in the province, and the transmissibility index was accurate.

According to the temporal distribution of COVID-19 in Jilin Province (Fig. 3), the imported cases in Jilin reached a peak on January 22, and decreased after January 23. Since January 31, the imported cases have remained at a low level. On January 23, the city of Wuhan was closed. At the same time, Jilin Province implemented measures involving screening and isolation for outsiders. This time coincided with the period of decline in imported cases, indicating that the above interventions had obvious effects. On January 31, 2020, the measures of having a flexible working system and fewer meetings were implemented. Personnel were required to wear masks when entering or leaving public places. From Fig. 3, we can see that since February 1, the number of secondary cases and daily actual incidence has been decreasing. Since January 31, 2020, the implementation time of intervention measures such as reducing travel and wearing masks has been consistent with the incidence decline time. This shows that the above intervention measures were effective during this period.

The National Health and Construction Commission of China analyzed more than 8400 cases that recovered and discharged. The clinical classification of these cases shows that the proportion of mild and normal cases is 90.8%, the proportion of severe cases is 7.2%, and the proportion of critical cases is 2% [37]. The mild and normal cases in Jilin Province accounted for 89.2% of all clinical classifications, which is consistent with the national clinical classification distribution [38]. This shows that most cases have mild symptoms and are as easily treated as patients with common influenza. For this reason, it has been difficult to investigate with whom infected persons have had close contact. Therefore, many sources of infection were not effectively isolated in the external environment in the early stage of the disease and in the early stage of the outbreak, which was the main reason for the public response delay in the early stage of the outbreak.

The age of onset of COVID-19 in Jilin was mainly between 20 and 59 years. Among these cases, people aged 30–49 years most commonly had mild and normal cases [39]. Therefore, among young adults and middle-aged persons, prognosis is better and mortality is low.

In this study, according to the time distribution characteristics of the epidemic curve of COVID-19 in Jilin Province, taking February 1st as the time cut-off point, the data were divided into two sections to fit the secondary cases; the fit was improved (*R*^2^ = 0.593, *P* < 0.001). According to the fit results, *R*_*eff*_ of the first stage (before February 1) was 1.64, indicating that the infection source of COVID-19 could infect approximately two people during the infectious period. If intervention had not occurred in time, allowing the disease to progress naturally, COVID-19 in Jilin would have continued to spread widely. The *R*_*eff*_ in the second stage (after February 1) was 0.05; that is, the infection source of a new coronavirus could infect 0.05 people during the infectious period, indicating that the epidemic situation had been controlled by this stage. The comprehensive intervention measures in Jilin Province reduced the transmissibility of COVID-19 by 96.95%.

Combined with a series of related measures since the outbreak in Jilin Province, a series of other measures were also launched on January 22, including closing tourist spots, suspending business operations, ensuring good sanitization and ventilation in public places, and banning trade in wild animals. On January 25, 2020, the Jilin provincial government launched a Public Health Events level-I emergency response, strengthened the investigation of non-native people and isolated non-native people at home, strengthened body temperature testing, implemented disinfection and sterilization measures, encouraged wearing of masks, and strengthened the management of large-scale activities. From January 31, 2020, the unit flexible working system was implemented to reduce the number of meetings and personnel input. The above measures were effective in the second stage of COVID-19, and transmissibility was reduced by 96.95%. Additionally, by the deadline (February 19), the actual number of secondary cases had been reduced by 92.31%, so that the pandemic was controlled. If Jilin had not taken measures and had allowed the disease to develop before February 1st, the prevalence of COVID-19 would have continued to spread in the province. The peak would have been reached by October 22, 2020, with the number of cases on that day being 177,011. The pandemic would have continued to be prevalent for 22 months, with a cumulative number of 17,129,367 cases, and an attack rate during the pandemic of 63.66%. Therefore, the series of prevention and control measures formulated and implemented in Jilin Province effectively controlled the progress of the COVID-19 pandemic, and, to the extent possible, helped avoid an interpersonal epidemic.

In the early stage of the outbreak, we developed a Bats-Hosts-Reservoir-People transmission network and assessed the human-to-human transmissibility of COVID-19 in Wuhan as 3.58 [21]. Studies have been conducted on the transmissibility of COVID-19 in different provinces and cities in China at different time periods, which found that the reproduction number ranged from 1.4 to 6.49, with a median of 2.79 in 12 studies [40]. Alimohamadi et al. used systematic reviews and meta-analysis to estimate the pooled *R*_0_ as 3.32 (95% CI, 2.81 to 3.82) [41]. Musa and others estimated that the *R*_0_ of COVID-19 in Africa was 2.37 [42]. Torres-Roman et al. estimated the overall basic reproductive number in Peru during the outbreak period was 2.97; Lima had a similar outcome, with an *R*_0_ of 2.88. Previous studies found that the transmissibility of COVID-19 in Jilin Province was lower than in other provinces and cities in China. Compared with densely populated cities, such as Wuhan, people living in Jilin Province have less contact with people, and higher social distance. This reduces the possibility of susceptible people contacting the infection; hence, the transmissibility in Jilin Province is lower than that in cities with higher exposure. This also illustrates the importance of isolation and increasing social distance. At the same time, due to geographical factors, the outbreak of COVID-19 was late to reach Jilin Province. The early outbreaks in cities such as Wuhan and Guangdong led to the accumulation of experience in responding to the outbreak by China’s health departments and the people. The people’s prevention and control measures were highly coordinated, and the health system responded quickly. As a result, compared with some European, African, and other countries, transmissibility in Jilin Province remained lower than that of other states. This shows that the earlier the medical system responds, the easier it is to control the spread of the outbreak. In the current study, we also found that most studies used the date of onset of confirmed cases to fit the model. However, because the data collection occurred at the beginning of the outbreak, there were some onset cases that had not been detected and reported. The incompleteness of the epidemic curve may cause *R*_0_ to become higher [41]. At the same time, the low early disease incidence and uneven quality of case reports may contribute to the difference in *R*_0_ [43], showing that the more complete the data when estimating the transmissibility of infectious diseases, the more accurate the research results.

## Limitations

The parameters in this research model were calculated based on the actual data of Jilin Province; therefore, data quality was high. However, the small number of actual cases would have affected the calculation of the model. There were only four asymptomatic infections in the data obtained, which reduced the reliability of the proportion estimation of asymptomatic infections in Jilin Province. At present, studies have shown that asymptomatic infections also have transmissibility. Such cases are not easy to find and isolate, which promotes the spread of disease and the outbreak. This model considered the effect of asymptomatic infection in the population. Therefore, error in the proportion of asymptomatic infections may have caused the prediction results to deviate from the actual situation.

In this study, the reciprocal of the incubation period calculated using the actual data of the COVID-19 spread in Jilin Province was a parameter in the model; thus, the accuracy of the incubation period calculation can also affect the model’s prediction. The incubation period of COVID-19 is 5–6 days [44], and the incubation period of the disease calculated in this study was 10 days in Jilin Province. The reason for this discrepancy may be that the time of contact with the first case is uncertain, and there are some cases with unclear contact time, such as repeated or continuous contact. Therefore, it is necessary to clarify the activity trajectory of secondary cases, or how long susceptible persons may infect others after being exposed to the source of infection. This is also a direction for exploration in future research.

In accordance with the epidemic trend of the disease, this study fitted the actual number of secondary cases in two stages. Additionally, the transmissibility of COVID-19 after February 1 was evaluated, and the effectiveness of preventive measures was verified. However, this study evaluated comprehensive prevention and control measures, but did not evaluate specific measures. It is not possible to determine which specific measures produced an effect. To solve this problem, it will be necessary to establish a model that considers individual prevention and control measures. However, the specific implementation time and completion status of each measure are difficult to determine, so this is likewise difficult to achieve.

## Conclusions

COVID-19 had moderate transmissibility in Jilin Province, China. The interventions implemented in the province were highly effective. The rapid response of the CDC and the health department, as well as increased social distancing and strict travel restrictions played a role in slowing or even controlling the outbreak. The sooner measures are taken, the faster the epidemic will decline. At present, the world is still in a stage in which the pandemic is not fully controlled. Therefore, relevant medical institutions should continue to strengthen prevention and control measures, and the specific measures for outbreak prevention and control in Jilin Province can be applied to other countries and regions.

## Data Availability

The datasets used and analyzed during the current study are available from Dr. Qinglong Zhao (jlcdczql@126.com) on reasonable request.

## Abbreviations

COVID-19: Coronavirus disease 2019
SEIAR: Susceptible – exposed – infectious – asymptomatic –recovered
